# Bipolar mood cycles associated with lunar entrainment of a circadian rhythm

**DOI:** 10.1038/s41398-018-0203-x

**Published:** 2018-08-13

**Authors:** Thomas A. Wehr

**Affiliations:** Intramural Research Program, National Institute of Mental Health, Bethesda, MD USA

## Abstract

In bipolar disorder, episodes of depression and mania are associated with dramatic disturbances in sleep, which experiments show are likely to contribute to the pathogenesis of the episodes. A recent finding that 18 patients’ manic-depressive cycles oscillated in synchrony with biweekly surges in amplitude of the moon’s tides provided a clue to the cause of the sleep-disturbances. Analyses of one of the patients’ sleep–wake cycles suggest that his mood cycles arose when a circadian rhythm that normally is entrained to dawn and controls the daily onset of wakefulness became entrained instead to 24.8-h recurrences of every second 12.4-h tidal cycle. The finding provides the basis for a comprehensive description of the pathogenesis and pathophysiology of the mood cycle.

## Introduction

In bipolar disorder, episodes of mania and depression are accompanied by dramatic changes in the timing and duration of sleep^1–4^. Sleep is abnormally long in depression and abnormally short in mania, and it is often completely absent during switches from depression to mania^4^. Experiments have shown that disrupting sleep of bipolar patients can induce mania and restoring sleep can induce depression^4–8^. It is thus likely that the sleep-disturbances are not only symptoms of the disorder, but also play a causal role in its pathophysiology. It also seems likely that identifying the cause of the sleep-disturbances would lead to a deeper understanding of the pathogenesis of the disorder and to new approaches to treatment.

A clue to the cause of the sleep-disturbances emerged from a recent finding that manic-depressive cycles in 18 patients with a rapid-cycling form of bipolar disorder oscillated in synchrony with biweekly surges in amplitude of the moon’s twice-daily gravitational tides^9,10^. This observation raised the question of how 12.4-h oscillations of the tidal cycle might give rise to multi-week cycles in sleep and mood. Since the timing and duration of sleep are regulated in part by the circadian pacemaker, one possibility is that one of the pacemaker’s components had uncoupled from the 24-h rhythm of the solar day and had become entrained to the 24.8-h rhythm of the lunar tidal day (the period of recurrence of every second tidal cycle). In this scenario, as the lunar-entrained rhythm went in and out of phase with solar-entrained rhythms, it would generate multi-week cycles in sleep and mood as it passed through phase-relationships with solar-entrained rhythms that experiments have shown would cause switches between depression and mania^2,4–10^.

To test that hypothesis, multi-year records of mood and sleep that had been maintained by one of the 18 patients were examined for evidence of interactions between 24-h solar cycles and 24.8-h lunar cycles^10^. It was predicted that (1) both solar and lunar periodicities would be detected in oscillations of the sleep–wake cycle; (2) the lunar periodicity would be most prominent during new-moon and full-moon surges in amplitude of the tidal cycle when its strength as a forcing cycle would be the greatest; and (3) the surges would coincide with switches between depression and mania. The results of those analyses are reported here.

## Patients and methods

### Patient characteristics

Details about the patient and the methods that were used to study his manic-depressive cycles were published previously without reference to lunar cycles^11^. In brief, he was a 51-year-old man who was diagnosed with the Structured Clinical Interview for DSM-III Axis I Disorders as having a bipolar disorder, rapid-cycling type. After the previous publication, his mood cycles were found to have oscillated in various modes of synchrony with the spring-neap cycle, a cycle in which the amplitude of the moon’s semi-diurnal gravitational tides reaches peak levels every 14.8 days when the sun, earth, and moon are aligned at the new moon and at the full moon. The focus of the analysis was the 1:2 mode of synchrony, a mode in which every mood cycle oscillated in synchrony with 29.5-day recurrences of every second spring-neap cycle^9^.

The patient gave written informed consent for his participation in the study, which was approved by the Institutional Review Board of the Intramural Research Program of the National Institute of Mental Health.

### Data acquisition and analysis

The patient used a 100-mm visual analog scale to record daily mood levels and a 24-h log-sheet to record daily times of sleep and wakefulness^11^.

To detect periodicities in the time series of sleep-wakefulness data, *χ*^2^ periodogram analyses were performed with the ActogramJ program^12,13^. Periods were sampled from 20.0 to 29.0 h, with the threshold for statistical significance set at *P* = 0.0001. Data from four different periods were analyzed separately: (1) a 101-day period during antidepressant treatment with nortriptyline + tranylcypromine when he kept to his habitual sleep-routine; (2) a 103-day period during antidepressant treatment with sertraline when he kept to his habitual sleep-routine; (3) a 122-day period during antidepressant treatment with sertraline when he was asked to sleep or be awake whenever he felt like doing so; and (4) a 100-day period during antidepressant treatment with sertraline when he was asked to rest and sleep in the dark on a regular schedule for 14 h (reduced gradually to 10 h) every night. Mood-stabilizer treatment with divalproex sodium was administered throughout.

### Data display

Conventional raster plots of sleep–wake cycles were constructed by dividing longitudinal records of their time series into 24-h (solar day) or 24.84-h (lunar tidal day) segments. The segments were then displayed sequentially beneath one another. The resulting array was then double-plotted to the right, to reveal the inherent continuity of the data and to facilitate visual inspection of the courses of the sleep–wake cycles across the plotting space. The two versions of the plots make it possible to view the behavior of the sleep–wake cycle in relation to the solar day and in relation to the lunar tidal day.

## Results

### Periodicities in the sleep–wake cycle

**Fig. 1:**
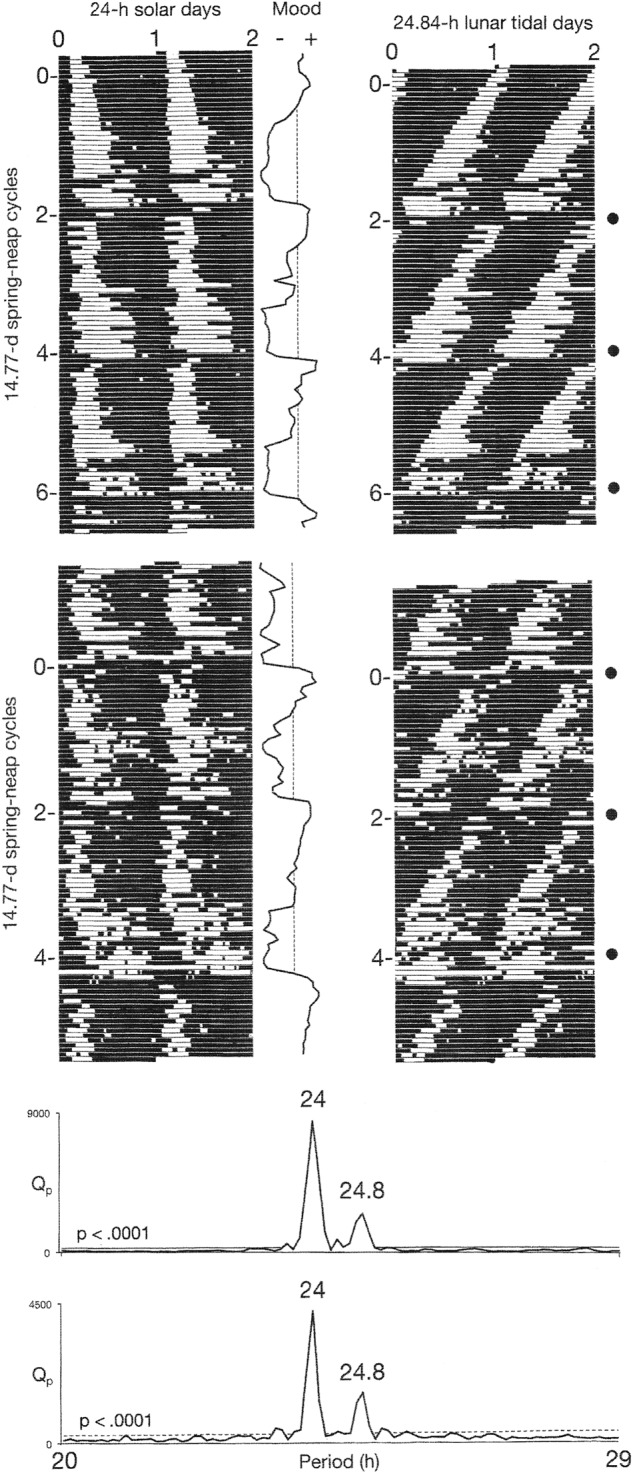
Bipolar mood cycles coincided with beat cycles that arose as wake-onsets entrained to the 24.8-h lunar tidal day went in and out of phase with sleep onsets entrained to the 24-h solar day. Periods of wakefulness (black horizontal bars) and sleep (white horizontal bars) are shown in a raster format. Raster plots and *χ*^2^ analyses of sleep–wake data are shown for two different treatment conditions (upper and lower), as described in the Data acquisition and analysis section. The 24-h periodicity (*p* < 0.0001) in the periodograms reflects the strong coupling of sleep onsets with the solar day. The 24.8-h periodicity (*p* < 0.0001) reflects the coupling of wake-onsets with the lunar tidal day. Phase-jumps in the timing of wake-onsets coincide with recurrences of new moons (filled circles) and with switches from depression (–) into mania (+). In the periodograms, Qp is a measure of variance in the data at each period tested. High variance indicates a strong periodicity. The dashed line shows the threshold for statistical significance of *p*  ≤ 0.0001

**Fig. 2:**
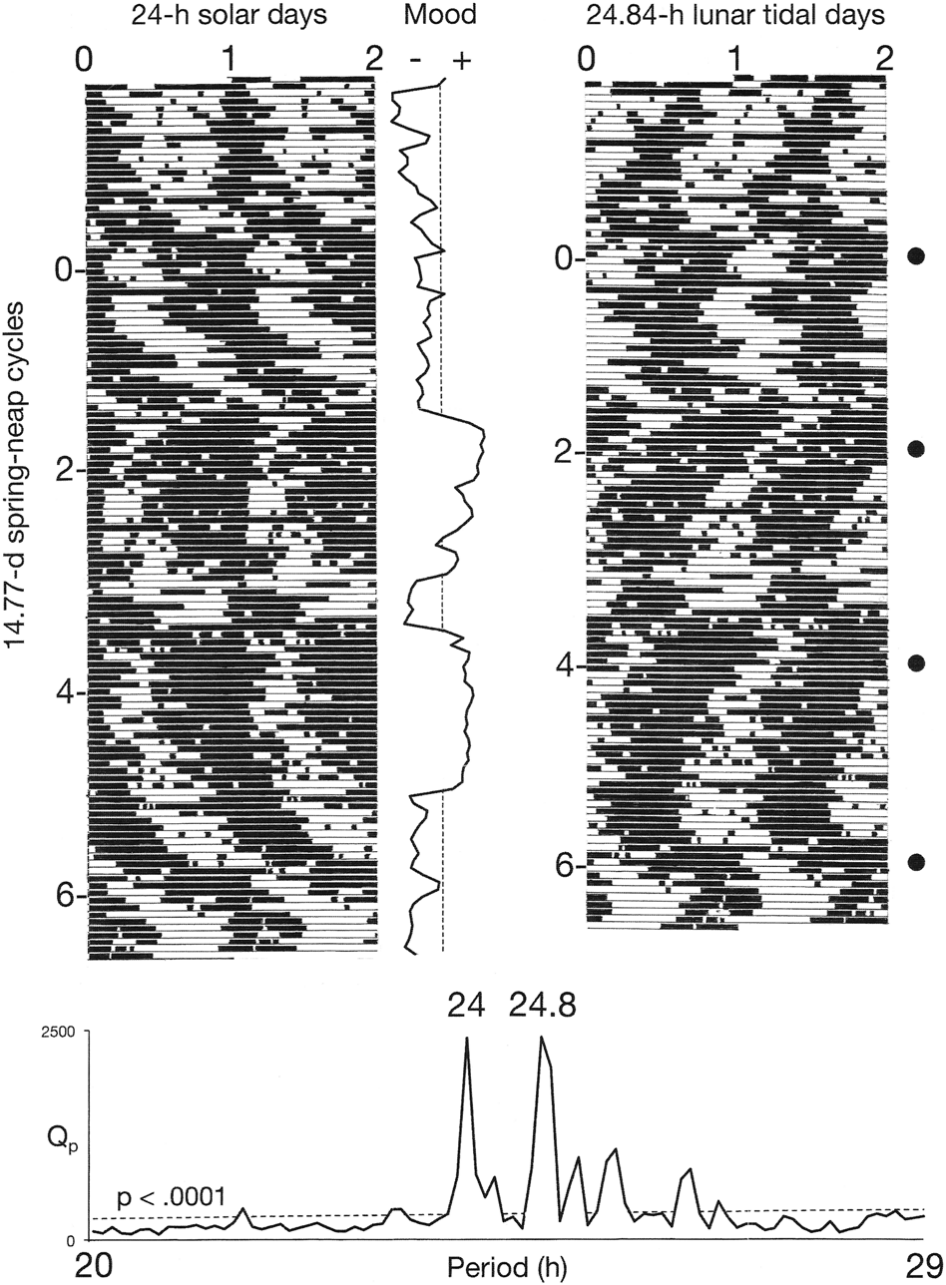
Coupling of the sleep–wake cycle to the 24.8-h lunar tidal day relative to its coupling to the 24-h solar day increased during an ad libitum sleep routine. Indications are the same as for Fig. 1. When the patient relaxed his adherence to a 24-h schedule, his sleep–wake cycle’s entrainment to the lunar tidal day and its relative coordination with the solar day were more clearly evident, and the 24.8-h lunar signal (*p* < 0.0001) and the 24-h solar signal in the periodogram became equal in strength

**Fig. 3:**
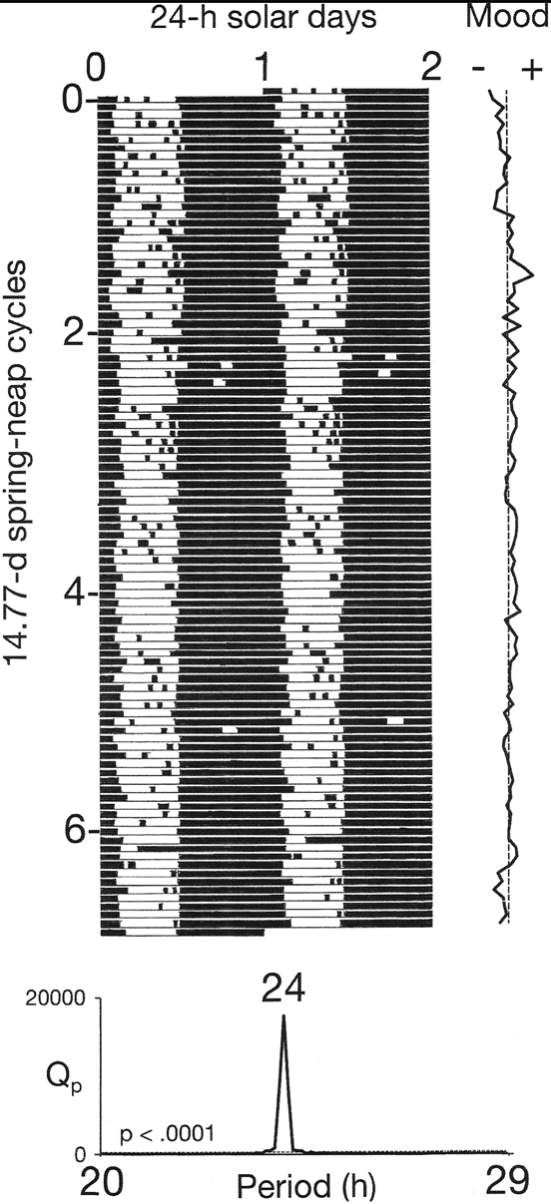
When the patient slept during long, rigidly scheduled dark periods, the lunar periodicity disappeared and his mood cycles stopped. Indications are the same as in Fig. 1

In the first three observation periods, *χ*^2^ periodograms detected robust periodicities that corresponded to the 24.0-h solar day (*p* < 0.0001) and the 24.8-h lunar tidal day (*p* < 0.0001) (Figs. 1 and 2). When the patient followed his habitual sleep-routine, the solar signal was stronger than the lunar one (Fig. 1). When he followed the ad libitum sleep-routine, the lunar signal and the solar signal were of equal strength (Fig. 2). When he adhered to a rigid schedule of rest and sleep during long periods of darkness every night, the lunar signal disappeared and his mood cycling stopped (Fig. 3).

### Associations of mood and sleep with a lunar phase

**Fig. 4:**
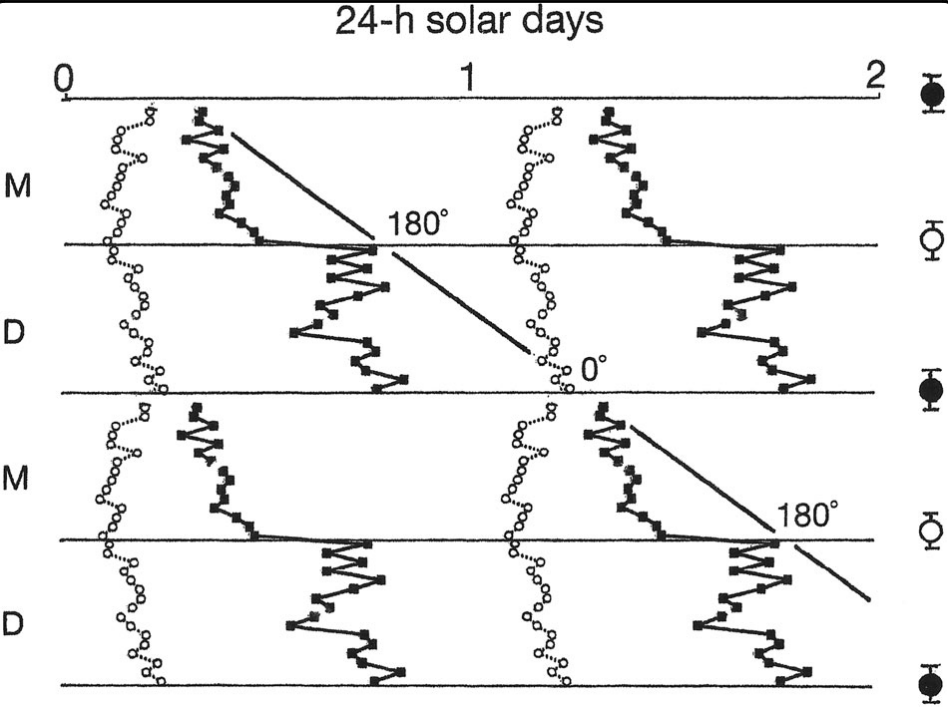
New-moon and full-moon surges in the tidal cycle’s strength as a forcing cycle were associated with 180° delay-type phase-jumps in wake-onset and with switches between depression and mania. Mean times of sleep-onset (small open circles) and wake-onset (small filled squares) during the 8 days that preceded and the 8 days that followed switches (horizontal lines) between mania (M) and depression (D) in Fig. 1 have been combined into a composite image based on the raster double-plot format of Fig. 1. Mean ± S.D. times of new moons (large filled circles) and full moons (large open circles) relative to the times of switches from depression to mania and switches from mania to depression, respectively, are shown to the right. Switches from mania to depression were defined as transitions from high mood ratings to the beginning of a rapid decline in mood ratings, and switches from depression to mania were defined as transitions from low mood ratings to the beginning of a rapid rise in mood ratings. The diagonal line, which approximates the passage of the 24.8-h lunar tidal day across the plotting space, is provided as a visual aid to highlight the fact that the phase-jumps caused wake-onset to shift 180° later from each switch to the switch that followed. The phase-jumps are interpreted as shifts between two metastable modes of coupling between wake-onset and sleep-onset

**Fig. 5:**
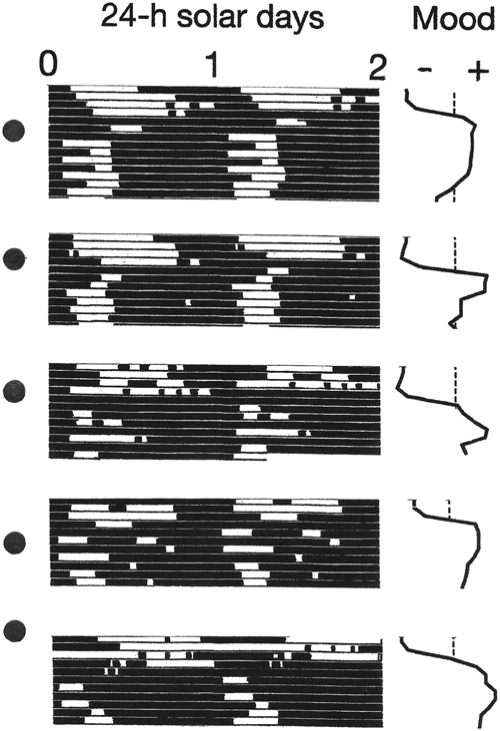
Nights of total insomnia accompanied switches from depression to mania. The display shows sections of raster plots from Fig. 1. Absence of sleep is interpreted to have arisen from a simultaneity between sleep-onset and wake-onset that occurred when new-moon surges in amplitude of the tidal cycle caused the phase position of wake-onset to jump from 180° to 0° relative to the phase-position of sleep-onset

Switches from depression into mania coincided with 29.5-day recurrences of the new moon, while switches from mania into depression coincided with 29.5-day recurrences of the full moon—times when the tidal cycle’s amplitude and its strength as a forcing cycle would have been the greatest (Figs. 1 and 4). Both types of switches were associated with dramatic delay-type phase-jumps in the timing of wake onsets (Fig. 4). The switches from depression into mania were also accompanied by one or more nights of total insomnia (Figs. 1 and 5).

## Discussion

As predicted, the analyses detected robust signals of both solar day and lunar tidal day periodicities in the oscillations of the patient’s sleep–wake cycle. Moreover, the two periodicities were consistently present in three different conditions of measurement comprising hundreds of sleep–wake cycles (Figs. 1 and 2). When the patient adhered to a rigid schedule of rest and sleep during long periods of darkness every night, the lunar signal disappeared and his mood cycling stopped (Fig. 3). In that schedule, exposure to the long periods of darkness may have stabilized his mood through their known capacity to increase the magnitude of the circadian pacemaker’s phase-resetting response to light and thereby to strengthen its coupling to the day–night cycle^14,15^. Taken together, these results support the hypothesis that interference between two components of the circadian system that were separately entrained to the solar day and the lunar tidal day generated the mood cycles.

Specific details in the results can be interpreted in light of what is known about the organization and structure of the circadian pacemaker. Pacemaker cells, which individually generate circadian rhythms in the suprachiasmatic nucleus of the hypothalamus, are organized in separate networks^16^. One network (E) is entrained to dusk and controls the timing of sleep-onset. Another network (M) is entrained to dawn and controls the timing of wake-onset. As the interval between dusk and dawn changes over the course of the year in a natural light environment, E and M make corresponding adjustments in duration of the interval between sleep-onset and wake-onset^17,18^. Under certain circumstances, the oscillations of E and M can become dissociated from one another. In this situation, coupling forces between the two components continue to operate and tend to draw the components into either of two metastable modes of coupling that are 180° apart^17,19^.

As is evident in Fig. 1, the 24-h periodicity in the chi-square periodograms primarily resulted from a strong coupling of sleep onset (E) to the solar day. In contrast, the 24.8-h periodicity appears primarily to have arisen from the behavior of wake onset (M), which exhibited coupling to both the solar day and the lunar tidal day, in a pattern of relative coordination in which the lunar cycle predominated.

As wake-onset went in and out of phase with sleep-onset, it appeared to do so by periodically executing 180° delay-type phase-jumps from one metastable mode of coupling with sleep-onset to the other (Fig. 4). Consistent with the hypothesis that wake-onset was entrained to the tidal cycle, the phase-jumps took place during recurrences of new-moon and full-moon surges in the tidal cycle’s strength as a forcing cycle. Consistent with the hypothesis that this entrainment drove the mood cycles, the phase-jumps invariably were accompanied by switches between depression and mania and by abrupt changes in the interval between sleep-onset and wake-onset (Fig. 4). In this way, the biweekly surges in amplitude of the tidal cycle acted like a pump that drove the mood cycle by causing ratchet-like 180° shifts in the phase-angle between E and M. The shifts were manifested in behavior as switches between depression and mania, and they caused sleep to be short in mania and long in depression.

As typically occurs in this disorder, the patient experienced one or more nights of total insomnia each time he switched from depression to mania (Figs. 1 and 5)^4,10^. The absence of sleep at those times can be attributed to a point of singularity in which sleep-onset and wake-onset occurred simultaneously after a phase-jump caused the phase-angle between them to shift from 180° to 0° (Fig. 4). Thereafter, an ongoing entrainment of wake-onset to the 24.8-h lunar cycle opened the phase-angle and produced the short periods of sleep that are characteristic of mania.

The results of experimental manipulations of sleep in bipolar disorder show that the changes in timing and duration of sleep that resulted from interactions between solar and lunar cycles could have caused the mood and behavioral changes that accompanied them. These results can be summarized as showing that being awake or being asleep during a circadian interval that normally coincides with sleep in the second half of the sleep period will determine whether the patient subsequently will be manic or depressed, respectively^2–8^. Based on these observations, one can infer that the cyclic changes in timing and duration of sleep that were caused by entrainment of wake-onsets to the lunar tidal day drove the mood cycle by causing the patient to alternate between sleep and wakefulness during this sleep-sensitive interval.

Halberg in 1967 was the first to hypothesize that a circadian rhythm could cause multi-week mood cycles if it became uncoupled from the day–night cycle and beat against other rhythms that were still entrained to the day–night cycle. In his model, he assumed that the decoupled rhythm would be free-running^20^. The model presented here attributes uncoupling of the rhythm from the day–night cycle to its entrainment to the lunar cycle, and it specifies sleep and wakefulness as mediators of the rhythm’s effects on mood. The association here between phase-jumps in the coupling mode of E with M, and switches between depression and mania, supports a circadian “bifurcation” hypothesis proposed by Kripke et al.^21^.

The role of dusk-tracking and dawn-tracking components of the circadian pacemaker in this model of pathogenesis establishes a link between rapid-cycling and seasonally cycling forms of a bipolar disorder. The link is based on the fact that in many types of animals, these same two components detect seasonal changes in duration of the photoperiod and regulate the timing of seasonal rhythms in their behavior^17^. Preliminary evidence indicates that these components may also regulate the timing of seasonal mood cycles in humans^22^. Thus, this patient’s rapid-cycling bipolar disorder can be viewed as a disorder of two networks in the suprachiasmatic nucleus that track seasonal changes in the photoperiod, a disorder in which a network that normally is entrained to dawn becomes entrained instead to the lunar tidal day. The episodes of depression and mania that occur in the rapid-cycling patient as the dawn-tracking component goes in and out of phase with the dusk-tracking component could then be viewed as seasonal behaviors that have been compressed into a truncated “year” of brief “winters” and brief “summers” with abrupt transitions between them.

A recent report that sporadic, non-seasonal episodes of bipolar depression respond to the type of light-treatment that is used to treat winter depression raises the possibility that most forms of bipolar disorder share a common neural substrate in the retinohypothalamic tract and the suprachiasmatic nucleus of the hypothalamus, and that the model of pathogenesis presented here might be applied more broadly^23^. In this regard, it is worth noting that state-dependent differences in the timing of wake-onset in this patient are consistent with recent findings in patients who were not specifically identified as rapid-cycling patients^24,25^.

Bipolar patients tend to be “evening types” who have a delayed phase-angle of entrainment to the day–night cycle^26^. A delayed phase-angle could occur if the intrinsic rhythm of pacemaker cells was abnormally slow, or if their response to the phase-resetting effect of light was abnormally weak. Either of these possibilities could be a risk factor for developing rapid-cycling by making a circadian rhythm susceptible to capture by the slower-than-24-h cycle of the lunar tidal day. This risk could be increased by the administration of certain antidepressants, which have been shown to slow the oscillations of the pacemaker and to interfere with its entrainment to light–dark cycles in experimental animals, and to cause rapid cycling in certain bipolar patients^27–29^.

The findings in this patient present a straightforward picture of pathogenesis that is much simpler than one might expect of a psychiatric disorder. They identify an external force that drives the mood cycle and the site and mechanism of its action in the brain. The findings account for most of the salient features of the disorder: its cyclicity and the frequencies in which it is expressed, the switch-like nature of transitions between depression and mania, the nights of total insomnia that accompany switches into mania, the hyposomnia of mania and the hypersomnia of depression, and the changes in mood in mania and depression that would be caused by the changes in sleep. Finally, the link between rapid and seasonal mood cycles indicates that the clinical features of mania and depression in rapid-cycling bipolar disorder may derive from seasonal adaptations of human behavior.

The nature of the mechanism that makes it possible for an object as small as the human body to be affected by the minute changes in gravity that are associated with lunar tidal cycles is unclear and is a question for future research. As a possible answer, Fisahn and colleagues have developed a model based on a quantum physical approach to the problem^30^. Although skepticism is warranted, lunar mood cycles may be an experiment of nature that is pointing toward aspects of gravity and biophysics that are only beginning to be investigated.
